# Book Reviews

**Published:** 1893-09

**Authors:** 


### *BOOK REVIEWS.*

---

A Text-Book of Medicine for Students and Practitioners. By Dr. Adolph Strümpell, Professor and Director of the *Medical Clinique* at Erlangen. Second American edition translated from the second and third, and revised from the sixth German edition by Herman F. Vickery, M. D., and Philip Coombs Knapp, M. D., with editorial notes by Frederick C. Shattuck, A. M., M. D. One hundred and nineteen illustrations. New York: D. Appleton & Co.

There is no text-book on practice that is more deservedly popular than that of Strümpell. From the time of its first appearance, some years ago, it has occupied a high place among works of its class, and the subsequent editions that have appeared have only increased its excellence and usefulness. For such a work the reviewer can give only the highest commendation.

The German work needed to be supplemented for American readers, and this has been wisely done by the translators. Thus, entire chapters on Dengue, Typho-malarial fever and Yellow fever were added because these diseases are not visitants of Germany. Under "Malarial Diseases" we note that the author makes no mention of Laveran's discovery of "plasmodium malariæ," and it remained for the translators to supply the omission. Under "Chronic Gastric Catarrh" the author lays some stress upon the examination of the contents of the stomach, and describes in detail the methods of so doing. Dr. Strümpell has great confidence in the development of Pasteur's inoculation for rabies. Cold bathing in the treatment of fevers is described and recommended. The chapters on diseases of the heart, and nervous diseases are possibly the most complete and practical in the entire work. An appendix on the symptoms and treatment in cases of poisoning closes the volume.

While Strümpell's work is full and complete, too much space is nowhere given to detail. The author appears to know as well what to leave out as what to put in. We think anyone who has this book will be highly pleased with it. Our own volume is used frequently and with great satisfaction.

A PRACTICAL TREATISE ON MATERIA MEDICA AND THERAPEUTICS, with Special Reference to the Clinical Application of Drugs. By John V. Shoemaker, A. M., M. D., Professor of Materia Medica and Clinical Medicine, and Clinical Professor of Diseases of the Skin in the Medico-Chirurgical College of Philadelphia; Physician to the Medico-Chirurgical Hospital, etc. Second Edition. In two volumes. Philadelphia: F. A. Davis Company, 1916 Cherry street.

The first volume of this work is devoted to Pharmacy, General Pharmacology and Therapeutics, and remedial agents not properly classed with drugs. In classifying the remedies of the Materia Medica the author adopts the arrangement of Brunton. Among the non-pharmacal remedies which are very well discussed are, electricity in medicine, massage and rest-cure, clinical applications of water, diet in disease, climatotherapy, hypnotism and suggestion, etc. Much space is devoted to the applications of electricity, and this portion of the author's work is probably the best. Even the therapeutic value of music is discussed.

The second volume is an "Independent Volume upon Drugs." The alphabetical arrangement of drugs is followed, which is convenient for reference, to say the least. With the exception of the descriptions of some of the newer drugs, such as Europhen, piperazin, methylene blue, etc., the volume contains nothing that is not found in other books. This volume is rather a disappointment when we note that it is written "with special reference to the clinical application of drugs." The use of animal extracts and juices is mentioned, especially the treatment of myxœdema by means of thyroid gland extract.

Dr. Shoemaker's contributions to medical literature have been both voluminous and various. From "Heredity, Health and Personal Beauty" to a two-volume Treatise on Materia Medica may be a wide interval, but his versatile genius embraces it all. Whatever may be said of his other works we do not think the present one a success.

---

### LITTELL'S LIVING AGE.

The contents of late numbers of *The Living Age* exhibit the usual wide range of subjects and nice discrimination in their selection characteristic of this leading eclectic magazine.

Foremost in value and interest is an article entitled "Literary Discoveries in Egypt." The land of Egypt is ever of deepest interest; around its name cluster memories of bygone ages, and from its bosom are drawn, from time to time, literary treasures of untold value. The article to which we have called attention gives a full account of numerous clay tablets, some of them made of Nile mud, covered with cuneiform inscriptions, unearthed within a few years at Tell el-Amarna, and which prove to be a correspondence between certain kings of Egypt who lived in the fifteenth century B. C., and their contemporaries and dependents. From these letters the reader will gain a fair insight into the social life of that time in Syria Babylon and Palestine.

Prominent articles of the issues of the present month are "The Chatham Islands and their Story," by Henry O. Forbes; "Aspects of Tennyson. Tennyson as a Nature-Poet," by Theodore Watts; "Our First Ambassadors to Russia," by Julian Corbett; "Fontainebleau" and "St. William of Norwich," by Augustus Jessopp. Scarcely second to these in interest are many others, notably "The Journal to Stella," by Austin Dobson; "The Influence of Climate on Race," by J. W. Fortescue; "Is the Universe Infinite," by Sir Robert Ball; "Addiscombe: The East India Company's Military College," by W. Broadfoot; "Some Thoughts on Pascal; "A Walk in Alexandria," by Alfred E. P. Raymund Dowling; and "Romance of the National Gallery," by Emily Constance Cook.

These numbers contain also some good short stories and poetry.

The subscription price of *The Living Age* is $8.00 a year, postpaid. A specimen copy may be had by sending fifteen cents to Littell & Co., Publishers, Boston, Mass.

---

### GRAY'S ANATOMY, NEW (13TH) EDITION.

Another edition, the thirteenth, of this standard work is announced for early publication by Messrs. Lea Brothers & Co. It is hardly too much to say that this work has been the most popular of all medical text-books whatever since its first appearance in 1851. Its text has been revised successively by the foremost anatomists of a generation, and the present edition embodies whatever changes were necessary to make it represent its advancing science. The illustrations have always been noted for their clearness. Their large size has rendered it possible to print the names of the parts upon them, thereby indicating not only their names, but also their extent—a most important matter. A liberal use of colors has been made to secure additional prominence for certain parts. Notwithstanding these improvements, the constantly increasing demand has justified a reduction in the price of the colored edition. An early review will appear in these columns.

